# Immune checkpoint‐related gene polymorphisms are associated with acute myeloid leukemia

**DOI:** 10.1002/cam4.6468

**Published:** 2023-08-21

**Authors:** Yuyan Wu, Mingying Li, Guangqiang Meng, Yuechan Ma, Jingjing Ye, Tao Sun, Chunyan Ji

**Affiliations:** ^1^ Department of Hematology Qilu Hospital of Shandong University, Cheeloo College of Medicine, Shandong University Jinan Shandong Province People's Republic of China; ^2^ Shandong Key Laboratory of Immunohematology Qilu Hospital of Shandong University Jinan Shandong Province People's Republic of China

**Keywords:** acute myeloid leukemia, immune checkpoint, PD1, prognosis, single nucleotide polymorphism, susceptibility

## Abstract

**Background:**

Chemotherapy is still the standard regimen for treating acute myeloid leukemia (AML) and its disappointing efficacy requires the urgent need for new therapeutic targets. It is well known that immune response plays an increasingly significant role in the pathogenesis of AML.

**Methods:**

We detected nine single nucleotide polymorphisms (SNPs) in immune checkpoint‐related genes, including PD1, LAG3, TIM3, and TIGIT in 285 AML inpatients and 324 healthy controls. SNP genotyping was performed on the MassARRAY platform. Furthermore, we analyzed the relationship between the susceptibility and prognosis of AML and the selected SNPs.

**Results:**

Our results showed that rs2227982 and rs10204525 in PD1 were significantly associated with susceptibility to AML after false discovery rate correction. PD1 rs10204525 also showed a significant correlation with the response to chemotherapy and risk stratification of AML. Importantly, the AA genotype of PD1 (rs2227982) under the recessive model showed a negative impact on AML prognosis independently.

**Conclusions:**

Our results indicate that PD1 SNPs are important for susceptibility and prognosis in AML, which may provide a new therapeutic target for AML patients.

## 1 INTRODUCTION

Acute myeloid leukemia (AML) is a hematopoietic malignancy characterized by uncontrolled proliferation of myeloid cells with arrested differentiation.^1^, ^2^ Although many targeted therapies, including FLT3 inhibitors and IDH inhibitors, are being researched or even in clinical trials, the treatment efficacy in AML patients is disappointing, and anthracycline‐ and cytarabine‐based chemotherapy regimens remains the first‐line treatments.^3^, ^4^ Therefore, there is an urgent need to explore new targets for treatment of AML.

Recent studies shed light on the function of the immune response in AML. The immune checkpoints controlling inhibition signal pathways mainly hamper T‐cell‐mediated immune responses.^5^, ^6^ Thus, aberrant expression of coinhibitory molecules may cause exhaustion of T cells, which fails to kill AML cells. The coinhibitory molecules consist of programmed death‐1 (PD1), lymphocyte activating 3 (LAG3), T cell immunoglobulin‐3 (TIM3) and T cell immunoglobulin and ITIM domain (TIGIT). PD‐1 is an inhibitor of T cell activation, engaged by PD‐L1. PD‐1 inhibitors have been used to reverse the exhaustion of the immune response in AML.^7^, ^8^ LAG‐3, TIM3, and TIGIT are mainly expressed in T cells, and their inhibitory function becomes evident upon induction of disease.^9^ However, immune therapy targeting only checkpoints is not sufficient and further study on the underlying mechanism is needed.^10^, ^11^

Single nucleotide polymorphisms (SNPs) are the most common type of genetic variants in humans and increasing research reports their important function in human diseases.^12^, ^13^ In AML, the AA genotype of interleukin‐13 (IL13) (rs1295686) increased the risk of AML, while IL8 (rs2227307) decreased the risk of AML.^14^ The GG genotype of rs6887695 of IL‐12A decreased the survival of AML patients.^15^ TNF‐α and IL‐10 are associated with the susceptibility to AML.^16^ However, the role of immunity‐related SNPs in susceptibility and prognosis has been uncovered. In this study, we explored the nine immunity‐related SNPs in 285 AML cases and 324 healthy controls and found that the SNPs of PD1 were associated with susceptibility to AML, risk stratification, survival, and response to anthracycline‐based induction chemotherapy.

## 2 MATERIALS AND METHODS

### 2.1 Patients and controls

Two hundred eighty‐five patients with newly diagnosed AML were recruited from Qilu Hospital of Shandong University from June 2011 to December 2021. All patients with a median age of 49 (13–83) years were diagnosed in accordance with the World Health Organization classification system and National Comprehensive Cancer Network (NCCN) guideline criteria. The chemotherapy response of AML was assessed according to the 2017 European Leukemia Network criteria. Accordingly, the control group included 324 healthy donors with a median age of 40 (20–88) years. The clinical characteristics of all participants including AML patients are demonstrated in Table 2. Patients who achieved complete response (CR) after induction chemotherapy were defined as CR. This study was approved by the Ethics Committee of Qilu Hospital, Shandong University as KYLL‐202206‐015‐1. All study participants were informed of consent in accordance with the Declaration of Helsinki.

### 2.2 DNA extraction and genotyping

Genomic DNA was extracted from mononuclear cells using DNA extraction kit (TianGen) according to the manufacturer's instructions. The concentration and purity of DNA were detected using a spectrophotometer. The SNPs of the selected genes are listed in Table 1. SNP genotyping was conducted using a mass spectrometry (MassARRAY) system (BGI Technology) based on time‐of‐flight and analyzed by polymerase chain reaction (PCR) amplification, single‐base extension reactions, resin desalination purification, and mass spectrometry. The genotyping was based on the previous reported methods.^15^

**TABLE 1:** Selected genes and SNPs.

Genes	SNPs
PD1	rs2227981
	rs10204525
	rs2227982
LAG3	rs2365094
	rs3782735
TIM3	rs1036199*
	rs10053538*
	rs10515746*
TIGIT	rs4682159*

Abbreviation: SNPs, single nucleotide polymorphisms.

* SNPs were not included in further analyses.

### 2.3 Real‐time PCR

RNA of AML patients with different genotypes was isolated with Trizol (Invitrogen) according to the manufacturer's instructions and reverse transcribed with reverse transcriptase. Real‐time PCR was conducted by mixing cDNA, primers and TB Green Premix Ex Taq™ (TAKARA). Amplification was performed on a LightCycler 480 II (Roche) according to the manufacturer's protocol. For each experiment, the expression data were normalized to the expression of GAPDH. The primer sequences were as follows: GAPDH, AAGGTGAAGGTCGGAGTCAAC and GGGGTCATT GATGGCAACAATA; PD1, CTCAGGGTGACAGAGAGAAG and GACACCAACCACCAGGGTTT.

### 2.4 Statistical analysis

All selected SNPs were tested for deviation from Hardy–Weinberg equilibrium (HWE) using Pearson's goodness‐of‐fit chi‐squared test. The relationships of the SNP genotype or allele frequency with the susceptibility to AML, risk stratification, CR and the survival analysis were conducted firstly using the chi‐squared test or Fisher's exact test for preliminary screening and then multivariate binary logistic regression analyses for the odds ratios (ORs) with corresponding 95% confidence intervals (95% CI) and *p*‐values adjusted for sex and age and Kaplan–Meier curves for estimating overall survival (OS). SPSS 26.0 software (SPSS Inc.) was used for statistical analyses. A two‐tailed *p* < 0.05 was considered statistically significant.

## 3 RESULTS

### 3.1 Study population

PD1, LAG3, TIM3, and TIGIT are the main immune checkpoints. Several inflammation factor‐related SNPs were selected (Table 1) and five SNPs were further analyzed after passing the HWE deviations and HapMap project test. The demographic and clinical characteristics of AML patients are shown in Table 2.

**TABLE 2:** Demographic and clinical characteristics.

Variable	AML patients
Sex (M/F)	150/135
Age (years), median (range)	49 (13–83)
WBC	
Median (×10^9^/L)	14.44
<100 × 10^9^/L	238 (83.5)
≥100 × 10^9^/L	47 (16.5)
PLT	
Median (×10^9^/L)	37
>50 × 10^9^/L	109 (38.2)
≤50 × 10^9^/L	176 (61.8)
HGB	
Median (g/L)	77
>60 g/L	239 (83.9)
≤60 g/L	46 (16.1)
Cytogenetics and molecular stratification	
Favorable prognosis, *n* (%)	93 (32.6)
Intermediate prognosis, *n* (%)	128 (44.9)
Adverse prognosis, *n* (%)	64 (22.5)
Response after the first course of anthracycline‐based induction therapy	
CR, *n* (%)	80 (61.1)
No CR, *n* (%)	51 (38.9)
Relapse after CR	
Yes, *n* (%)	18 (22.5)
No, *n* (%)	62 (77.5)

### 3.2 Association between SNPs and susceptibility to AML

We used four genetic models (co‐dominant, dominant, recessive, and allele) to analyze the associations between the selected susceptibility to AML and SNPs. Preliminary screening with the chi‐squared test or Fisher's exact test showed that the genotypic frequencies of SNPs rs2227981, rs10204525, and rs2227982 in the PD1 gene (co‐dominant, recessive, and allele models) were significantly associated with susceptibility to AML (*p* < 0.05) and no significant association between the genotypic frequencies of SNPs in the LAG3 gene (co‐dominant, recessive, and allele models) and the susceptibility to AML (*p* > 0.05) was found. Then, univariate logistic regression analysis was conducted to reveal the associations of the following SNPs of PD1 with susceptibility to AML: rs10204525 under recessive and allele models (*p* < 0.05) and rs2227982 under the co‐dominant, recessive, and allele models (*p* < 0.05) after adjusting for sex and age. However, after false discovery rate (FDR) correction, the allele model of rs10204525 and co‐dominant, recessive, and allele models of rs2227982 in PD1 were found to be associated with AML susceptibility (*p* < 0.05) (Table 3).

**TABLE 3:** Association between SNPs and susceptibility to AML.

Gene	SNP	Model	Genotype/allele	Cases (*n*)	Controls (*n*)	OR (95% CI)	Adjusted *p*‐value
PD1	rs2227981	Co‐dominant	GG	134	180		
			AA	27	30	1.044 (0.581–1.876)	0.887
			GA	124	114	1.376 (0.969–1.953)	0.074
		Recessive	GG	134	180		
			AA/GA	151	144	1.304 (0.937–1.815)	0.115
	rs10204525	Co‐dominant	CC	32	28		
			TT	115	164	0.560 (0.313–1.001)	0.05
			TC	138	132	0.923 (0.518–1.646)	0.787
		Recessive	TT	115	164		
			CC/TC	170	160	0.712 (0.511–0.993)	0.046
		Allele	T	368	460		
			C	202	188	1.428 (1.112–1.833)	0.005
	rs2227982	Co‐dominant	AA	21	75		
			GG	88	85	3.839 (2.123–6.942)	0.000
			GA	176	164	4.258 (2.449–7.402)	0.000
		Recessive	AA	21	75		
			GG/GA	264	249	4.109 (2.402–7.030)	0.000
		Allele	A	218	314		
			G	352	334	0.675 (0.533–0.854)	0.001

Abbreviations: CI, confidence interval; OR, odds ratio; SNP, single nucleotide polymorphism.

### 3.3 Associations between WBC, hemoglobin, PLT, and SNPs

As clinical characteristics may be related to SNPs, we analyzed the association of the selected SNPs with WBC, hemoglobin, and PLT of AML patients using the chi‐squared test or Fisher's exact test. In this study, WBC count 100 × 10^9^/L or lower was defined as low WBC count, and WBC count higher than 100 × 10^9^/L was defined as high WBC count. HGB 60 g/L or lower was defined as low HGB and HGB higher than 60 g/L was defined as high HGB. PLT 80 × 10^9^/L or lower was defined as low PLT and PLT higher than 80 × 10^9^/L was defined as high PLT. However, we found no such association with any of them (*p* > 0.05).

### 3.4 Association between SNPs and risk stratification of AML

Molecular genetic and cytogenetic mutations play an important role in risk stratification which guides the AML prognosis. According to NCCN guidelines, the recruited AML patients were categorized into three groups, including favorable, intermediate, and adverse prognosis groups based on karyotypic and molecular abnormalities. Then, the association between the selected SNPs and risk stratification was analyzed. Logistic regression analysis after adjusting for sex and age revealed that the co‐dominant and dominant model of rs10204525, the co‐dominant and recessive model of rs2227982 in PD1, and the recessive model of rs2365094 in LAG3 were associated with the prognosis of AML. After FDR correction, the co‐dominant CC genotype and the dominant CC genotype of rs10204525 were found to be associated with risk stratification significantly, with an increase in poor prognosis (*p* = 0.015) (Table 4), while no significant association between the recessive model of rs2365094 in LAG3 and the prognosis of AML (*p* > 0.05) was found.

**TABLE 4:** Association between risk stratification in AML and SNPs.

Gene	SNPs	Model	Genotype/allele	Favorable prognosis	Intermediate prognosis	Adverse prognosis	Adjusted *p‐*value OR (95% CI)
PD1	rs10204525	Co‐dominant	CC	4	16	12	0.005
							2.872 (1.373–6.013)
			TT	33	59	23	0.316
							1.270 (0.795–2.028)
			TC	55	51	32	
		Dominant	CC	4	16	12	
			TT/TC	88	110	55	0.009
							2.557 (1.264–5.176)
	rs2227982	Co‐dominant	AA	4	8	9	0.013
							2.983 (1.255–7.092)
			GG	26	35	27	0.067
							1.570 (0.969–2.547)
			GA	62	83	31	
		Recessive	AA	4	8	9	
			GG/GA	88	118	58	0.029
							2.565 (1.099–5.995)
LAG3	Rs2365094	Recessive	CC	7	29	12	
			GG/CG	85	97		0.04
							1.850 (1.028–3.330)

Abbreviations: CI, confidence interval; OR, odds ratio; SNPs, single nucleotide polymorphisms.

### 3.5 Associations between SNPs and the response to anthracycline‐based induction chemotherapy in AML patients

A previous study reported that anthracyclines could potentiate antitumor immunity, including relieve tumor induced immunosuppression and enhancing immune effector cell activation.^17^ As checkpoint was the important marker of immunosuppression,^6^ we conducted analysis to detect the association between SNPs and AML patient's response to anthracyclines. After analyzing the 243 patients with non‐M3 AML, 131 (53.9%) received anthracycline‐based induction chemotherapy. After the first course of chemotherapy, 80 (61.1%) patients achieved CR. Preliminary screening with the chi‐squared test or Fisher's exact test and the following univariate logistic regression analysis adjusting for sex and age showed that rs10204525 in PD1 was significantly correlated with response to chemotherapy under the recessive, co‐dominant, and allele models (*p* < 0.05). After FDR correction, the TT genotype under the co‐dominant and the recessive model and the T allele of rs10204525 were found to be associated with response to chemotherapy significantly, with an increase in CR (Table 5).

**TABLE 5:** Association between response after the first course of anthracycline‐based induction chemotherapy in AML and SNPs.

Gene	SNPs	Model	Genotype/allele	CR (*n*)	No CR (*n*)	OR (95% CI)	Adjusted *p*‐value
PD1	rs10204525	Co‐dominant	CC	7	10		
			TT	41	16	0.252 (0.081–0.791)	0.018
			TC	32	26	0.559 (0.183–1.705)	0.307
		Recessive	TT	41	15		
			CC/TC	39	36	0.395 (0.187–0.833)	0.015
		Allele	T	114	56		
			C	46	46	2.042 (1.214–3.434)	0.007

Abbreviations: CI, confidence interval; OR, odds ratio.

### 3.6 Associations between SNPs and relapse‐free survival

After anthracycline‐based chemotherapy, 122 patients achieved CR, but seven of them relapsed during treatment or follow‐up. We also analyzed the association of the selected SNPs with relapse‐free survival using the chi‐squared test or Fisher's exact test but found no such association with any of them (*p* > 0.05).

### 3.7 Associations between SNPs and survival in AML

**FIGURE 1:**
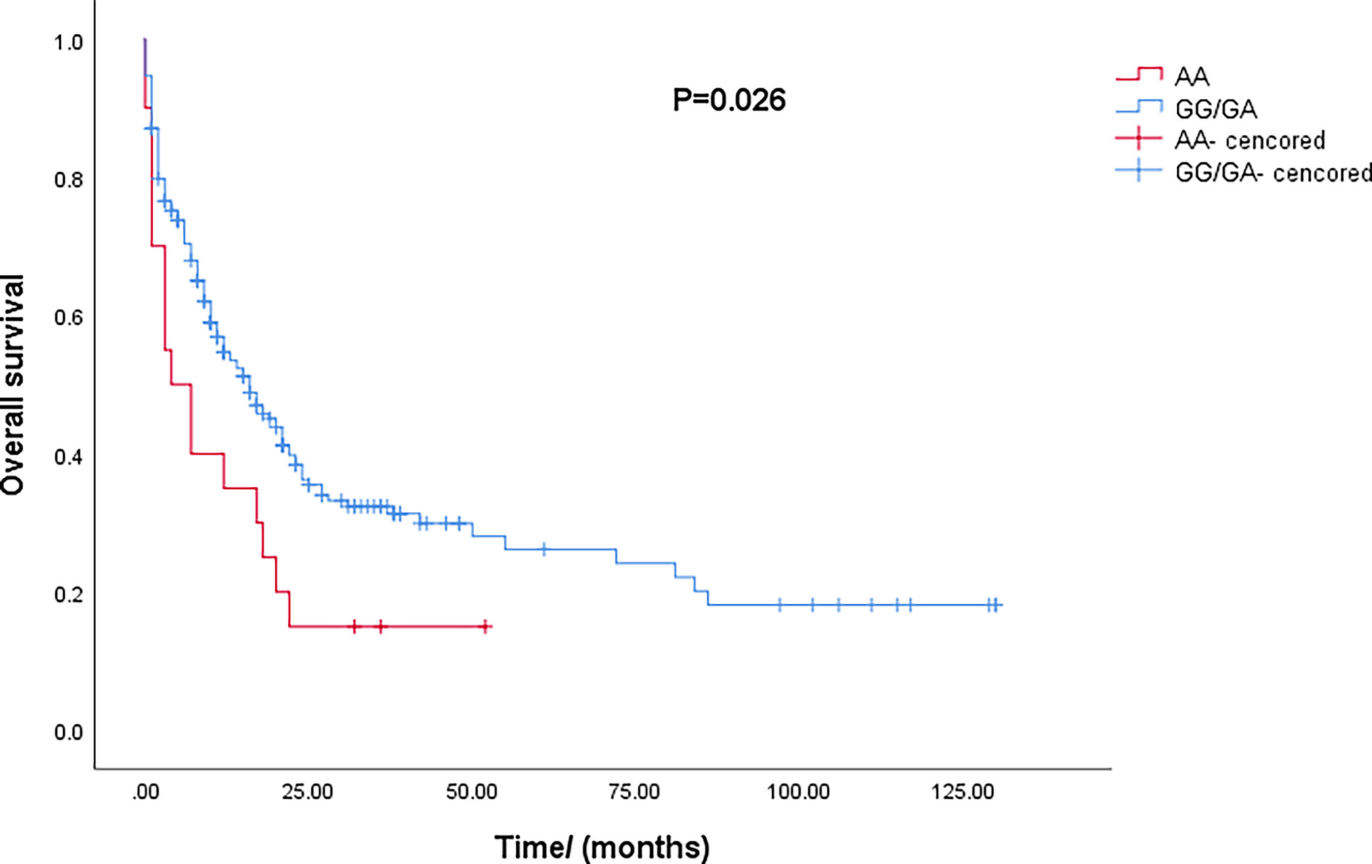
The overall survival of acute myeloid leukemia patients carrying the AA and GG/GA genotypes in rs2227982.

We used four models to analyze the relationships between the selected SNPs and the survival of non‐M3 AML patients. The preliminary screening of Kaplan–Meier analysis revealed that rs2227982 in PD1 was associated with prognosis under the recessive model (*p* < 0.05). For rs2227982 in PD1, the OS that AML patients with the AA genotype achieved was significantly shorter than the patients with the GG/GA genotypes achieved under the recessive model (Figure 1).

Cox proportional hazards model with multivariate analysis for survival was conducted to analyze rs2227982 of PD1, age, and risk stratification. In our study, intermediate prognosis, adverse prognosis, and age ≥60 years were able to negatively affect OS in the analysis independently. Under the recessive model, the OS of the patients with the AA genotype of PD1 rs2227982 was significant shorter than that of patients with the genotype GG/GA (OR = 1.872, 95% CI = 1.126–3.111). These results indicated that the AA genotype of PD1 rs2227982 was an independent unfavorable factor of AML prognosis.

### 3.8 PD‐1 mRNA expression in AML patients

**FIGURE 2:**
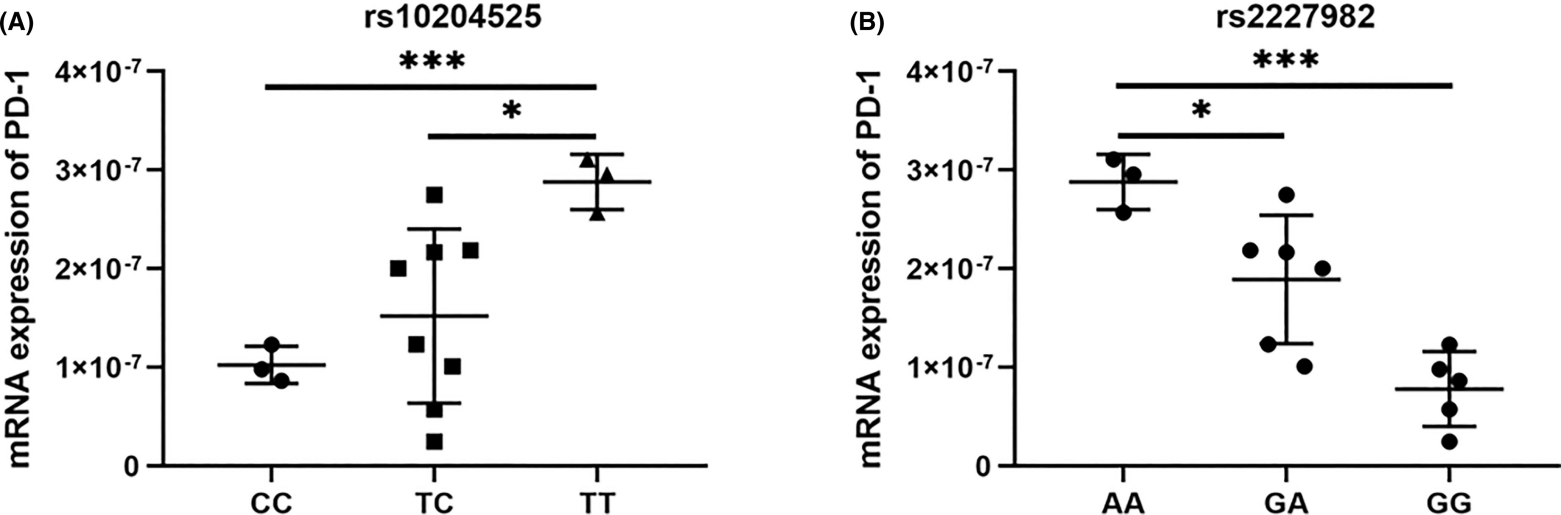
PD1 mRNA expression in acute myeloid leukemia (AML) patients. (A) Expression of PD1 mRNA in AML patients with the CC, TC, and TT genotypes of rs10204525. *n* = 3, 8, and 3, respectively. (B) Expression of PD1 mRNA in AML patients with the AA, GA, and GG genotypes of rs2227982. *n* = 3, 6, and 5, respectively. *p < 0.05, ***p < 0.001.

To validate the association between PD1 rs10204525 and rs2227982 in PD1 and the susceptibility toAML, we detected the mRNA expression of PD1 in 14 patients and found that the mRNA expression of PD1 in AML patients with TT genotype in rs10204525 was higher than that of patients with the CC and TC genotypes (Figure 2A). The result confirmed that the higher expression of the TT genotype of PD1 rs10204525 would be the reason for the susceptibility to AML, the response to chemotherapy, and risk stratification of AML. Meanwhile, the expression of AA genotype in PD1 rs2227982 was higher than that of patients with the GG and GA genotypes (Figure 2B). The result suggested that the higher expression of the AA genotype of PD1 rs2227982 could be the reason for the susceptibility to AML and the poor prognosis.

## 4 DISCUSSION

Here, we examined the association between immune checkpoint‐related gene (PD1, LAG3, TIM3, and TIGIT) polymorphisms and susceptibility, clinical variables, risk stratification, response to chemotherapy, relapse, and survival of AML. After strict statistical analysis, rs2227982 was significantly correlated with susceptibility and survival of AML and rs10204525 was significantly correlated with susceptibility, response to chemotherapy, and risk stratification of AML. However, no checkpoint‐related SNP contributed to the clinical variables and relapse of AML. These results indicate that PD1 may be related to the pathogenesis and progression of AML, which provides new targets for its diagnosis and treatment.

Although many studies have focused on prognosis, few reports have focused on susceptibility in AML. PD1 has attracted attention in many autoimmune diseases.^18^, ^19^ Mice with PD1 deficiency tended to suffer lupus‐like arthritis and glomerulonephritis.^20^ In addition, there are associations between PD1 SNPs and susceptibility to ITP and autoimmune hepatitis.^21^, ^22^ Blockade of PD1 has been reported to restore the antitumor response of T cells and relieve the leukemia burden.^23^, ^24^ In our study, we found that the C allele of rs10204525, the co‐dominant GG and GA, the recessive AA, and the G allele of rs2227982 in PD1 were associated with susceptibility to AML. The results suggested that rs2227982 and rs10204525 in PD1 are risk factors for AML susceptibility, providing new evidence of the impact of immune checkpoints on AML.

Many gene polymorphisms are reported to be associated with the response to chemotherapy and targeted therapy in AML patients. Drug resistance and cell apoptosis‐related genes were associated with the rate of CR, including genes encoding influx and efflux transporters.^25^, ^26^, ^27^ In addition, cytogenetic genes for risk stratification were important in predicting the outcome of chemotherapy.^28^, ^29^ However, the role of checkpoint‐related SNPs in the response to chemotherapy in AML remains unclear. Our results showed that the co‐dominant TT genotype, the recessive TT genotype, and the T allele of rs10204525 significantly increased the rate of complete remission, filling in relevant gaps.

Previous studies have shown that there are associations between SNPs and the prognosis of AML.^30^, ^31^, ^32^ The association between SNPs related to inflammation factor and AML has also been confirmed. Researchers have found that SNPs of NLRP3 (nucleotide‐binding domain‐like receptor family pyrin domain containing protein 3), IL‐18, and IL‐1β were associated with survival of AML.^33^ Our studies identified that blockade of NLRP3 restored the antitumor response by reducing the proportion of T cells expressing PD‐1, thus confirming the association between inflammasome‐related genes and immune checkpoints.^34^ In solid tumors, PD1 rs2227982 and rs10204525 are associated with the prognosis.^35^, ^36^, ^37^ However, the association between checkpoint‐related SNPs and survival in AML remains unclear. Our research found that rs2227982 in PD1 was significantly associated with the survival of AML patients in the recessive model, suggesting that this SNP locus has important reference value for predicting AML prognosis.

It has been reported that the role of PD1 expression was complicated in diseases. For example, Deipolyi et al. revealed that breast cancer patients with more PD1+ tumor‐infiltrating lymphocytes tended to achieve CR.^38^ However, the relationship between PD1 expression and poor prognosis was also found.^39^ The analysis of CR rate only assessed the patients whether achived CR after the first course of the chemotherapy, but the prognosis was affected by many factors, including drug resistance and severity of disease, not only treatment response. The specific impact mechanism of PD1 expression on different treatment stages needs to be verified through extensive experiments.

In summary, we found that rs10204525 and rs2227982 of PD1 might be important genetic markers correlated with the pathogenesis, treatment response, and prognosis of AML. Our findings provide new insight into therapeutic targets and prognosis prediction of SNPs in AML patients.

## AUTHOR CONTRIBUTIONS

**Yuyan Wu:** Data curation (equal); formal analysis (lead); investigation (equal); methodology (equal); resources (equal); software (equal); validation (equal); visualization (equal); writing – original draft (lead); writing – review and editing (equal). **Mingying Li:** Investigation (equal); methodology (equal); resources (equal). **Guangqiang Meng:** Investigation (equal); methodology (equal); resources (equal); software (equal). **Yuechan Ma:** Investigation (equal); methodology (equal); resources (equal); software (equal). **Jingjing Ye:** Conceptualization (equal); funding acquisition (equal); project administration (equal); supervision (equal). **Tao Sun:** Conceptualization (equal); funding acquisition (equal); project administration (equal); supervision (equal); writing – review and editing (equal). **Chunyan Ji:** Conceptualization (equal); funding acquisition (equal); project administration (lead); supervision (lead).

## CONFLICT OF INTEREST STATEMENT

The authors declare no competing interests.

## Data Availability

All data are available from the corresponding author on reasonable request. Source data are provided with this paper.
